# The Renaissance of KRAS Targeting in Advanced Non-Small-Cell Lung Cancer: New Opportunities Following Old Failures

**DOI:** 10.3389/fonc.2021.792385

**Published:** 2021-12-23

**Authors:** Miriam Grazia Ferrara, Alessio Stefani, Sara Pilotto, Carmine Carbone, Emanuele Vita, Mariantonietta Di Salvatore, Ettore D’Argento, Ileana Sparagna, Federico Monaca, Giustina Valente, Antonio Vitale, Geny Piro, Lorenzo Belluomini, Michele Milella, Giampaolo Tortora, Emilio Bria

**Affiliations:** ^1^ Comprehensive Cancer Center, Fondazione Policlinico Universitario Agostino Gemelli IRCCS, Roma, Italy; ^2^ Section of Oncology, Department of Translational Medicine, Università Cattolica Del Sacro Cuore, Roma, Italy; ^3^ Section of Oncology, Department of Medicine, University of Verona School of Medicine and Verona University Hospital Trust, Verona, Italy

**Keywords:** KRAS, lung cancer, mutations, precision medicine, targeted therapy

## Abstract

Non-small cell lung cancer (NSCLC) represents the perfect paradigm of ‘precision medicine’ due to its complex intratumoral heterogeneity. It is truly characterized by a range of molecular alterations that can deeply influence the natural history of this disease. Several molecular alterations have been found over time, paving the road to biomarker-driven therapy and radically changing the prognosis of ‘oncogene addicted’ NSCLC patients. Kirsten rat sarcoma (KRAS) mutations are present in up to 30% of NSCLC (especially in adenocarcinoma histotype) and have been identified decades ago. Since its discovery, its molecular characteristics and its marked affinity to a specific substrate have led to define KRAS as an undruggable alteration. Despite that, many attempts have been made to develop drugs capable of targeting KRAS signaling but, until a few years ago, these efforts have been unsuccessful. Comprehensive genomic profiling and wide-spectrum analysis of genetic alterations have only recently allowed to identify different types of KRAS mutations. This tricky step has finally opened new frontiers in the treatment approach of KRAS-mutant patients and might hopefully increase their prognosis and quality of life. In this review, we aim to highlight the most interesting aspects of (epi)genetic KRAS features, hoping to light the way to the state of art of targeting KRAS in NSCLC.

## Introduction: KRAS Mutations

Non-small cell lung cancer (NSCLC) represents one of the most multifaceted tumor based on its complex molecular features. The identification of driver mutations, predominantly identified in the adenocarcinoma histotype, have opened the era of ‘biomarker-driven therapy’ (1). The ‘oncogene addicted’ disease, characterized by a targetable gene alteration, represents a subgroup of NSCLC in which targeted agents have radically changed prognosis and quality of life of these patients (2). With the advent of tumor genotyping, upfront routine testing for targetable biomarker alterations is strongly recommended in all patients with diagnosis of NSCLC to personalize treatment (3). Additional molecular analysis by next generation sequencing (NGS) may be considered as part of broader testing panels, with the final aim of detecting rare druggable alterations to enroll patients in clinical trials. Among gene alterations involved in cancer development, kirsten rat sarcoma (KRAS) represents the most common oncogene driver in human cancer. It has been demonstrated that the wild-type KRAS is a proto-oncogene that is frequently activated during many types of cancer progression (4). Once the KRAS gene mutates, it acquires oncogenic properties that are causally involved in the development of human cancers (5, 6). Such oncogenic forms of the KRAS gene are prevalent in pancreatic carcinomas (>80%), colon carcinomas (40–50%), and lung carcinomas (30–50%), but are also present in biliary tract malignancies, endometrial cancer, cervical cancer, bladder cancer, liver cancer, myeloid leukemia and breast cancer (7). KRAS protein is a small guanine triphosphatase (GTPase) that serves as a binary switch in signal transduction for most receptor tyrosine kinases and plays a key role in regulating cell growth, differentiation and apoptosis by interacting with multiple effectors including mitogen-activated protein kinase (MAPK) (8). Oncogenic KRAS mutations mostly occur in exon 2 at codon 12, less frequently at codon 13 (3-5%) and rarely at exon 3 codon 61 (less than 1%) (9). KRAS signaling begins with the stimulation of a vast array of upstream receptors including receptor tyrosine kinases (RTKs). Adaptor proteins interact with the intracellular domain of activated EGFR and in turn recruit guanine nucleotide exchange factors (GEFs), such as Son of Sevenless (SOS), to the cellular membrane where they can associate with KRAS to promote the switch from inactive GDP to active GTP. KRAS signaling terminates upon the hydrolysis of GTP to GDP by the intrinsic GTPase activity of RAS. Cancer-causing mutations in KRAS drastically impair the GTPase activity, resulting in KRAS proteins that are locked in the active GTP-bound conformation, regardless of the upstream signal. As consequence, these missense mutations cause a constitutive activation of KRAS downstream pathways and promote cell proliferation and tumor development (10). In this review we aim to give an overview on KRAS role in lung cancer pathogenesis and targeting with a special focus on new potential treatments based on epigenetic modulation.

## KRAS in Lung Cancer: Clinical Features and Therapeutical Implications Before the Advent of Direct Inhibitors

In NSCLC, KRAS mutations occur in 30% of adenocarcinoma and less frequently (about 7%) in squamous-cell lung carcinoma (11). Nevertheless, more recent results suggest that, in most cases, KRAS mutations do not occur in pure squamous-cell lung carcinoma but in mixed histology of adenosquamous or neuroendocrine carcinomas (12, 13). Although most of KRAS-mutant NSCLC are diagnosed in former or active smokers, KRAS mutations can also be detected in never smoker patients with early onset of cancer, thus its mutational state cannot be predicted on the basis of smoking history alone (14). Because of its high frequency in lung adenocarcinoma, several preclinical and clinical investigations have been conducted seeking effective therapeutic approaches targeting KRAS mutations but, for many years, these efforts have been unsuccessful. The deeper knowledge of genomic alterations has led to the identification of different KRAS subtype mutations. Indeed, it has been demonstrated that in NSCLC the most frequent KRAS allelic variations are the p.G12C (39%) and p.G12V (17-18%), resulting from a G:C to T:A transversion as a classical smoking-induced alteration (15). Remarkably, smokers and never smokers have a different spectrum of mutations and codon variants in KRAS. Thus, transition mutations (G12D) are more common in never smokers, whereas transversion mutations (G12C and G12V) are more common in former or current smokers (16). Moreover, KRAS-mutant NSCLC in smoker patients are genomically more complex, with a higher mutational burden and higher frequency of additional mutations in TP53 or STK11 genes compared to never smoker tumors, as result of antigenic exposure and oxidative stress in epithelial cells (17). From a clinical point of view, KRAS-mutant cancers have generally been associated with poorer overall survival (OS) compared to KRAS wild type tumors, especially in the advanced stages (18–20); however, other studies in early (where the benefit of adjuvant chemotherapy is minimal) or advanced stage of KRAS-mutant lung cancer have provided conflicting results, thus the prognostic value of KRAS alteration is still debated (21–23). A systematic review and meta-analysis including 3,620 patients has shown that KRAS mutations confers a significantly worse prognosis in patients with lung adenocarcinoma (19). Furthermore, KRAS mutations also have a predictive value in lung cancer. Indeed, as already outlined, KRAS represents a critical downstream effector on the EGFR pathway. Thus, there is a biologic rationale supporting the hypothesis that KRAS-mutant NSCLC are intrinsically resistant to EGFR TKIs because of a constitutive activation of the kinase that regulates EGFR signaling (24–27). With this regard, several studied have evaluated the efficacy of erlotinib or gefitinib according to KRAS mutational status confirming an association between KRAS mutations and limited activity of EGFR TKIs (28, 29). Several investigators have also hypothesized that KRAS may be a predictive biomarker of response to chemotherapy. Indeed, early preclinical data have shown that KRAS mutations correlate with a greater sensitivity to pemetrexed in NSCLC models (30) probably due to the pemetrexed ability to alter KRAS RNA expression. From a biologic point of view, this result is explained by a strong dependency of KRAS-mutant cells on folate metabolism pathways. Unfortunately, these preclinical results did not translate into an increased clinical activity (31, 32); indeed, a retrospective study conducted on 1,190 KRAS-mutant NSCLC patients treated with upfront platinum-based doublet chemotherapy has demonstrated that pemetrexed was associated with a shorter progression-free interval compared to other agents, such as taxanes (32). Nevertheless, at the ESMO 2021 congress, in the NVALT22 study cisplatin-pemetrexed chemotherapy plus bevacizumab did not improve PFS compared with carboplatin-paclitaxel plus bevacizumab in KRAS mutant patients (33). Concerning other potential targets, Mainardi et al. (34) have found that Src homology region 2 (SHP2), a cytoplasmic Src homology 2 domain containing protein tyrosine phosphatase that regulates several cellular processes, is necessary for KRAS-mutant tumor cell growth in *in vivo* models of NSCLC. Accordingly, the authors have highlighted that SHP2 inhibition promotes a senescence response in KRAS-mutant NSCLC cells, suggesting a rationale for targeting SHP2 in NSCLC harboring KRAS mutations. Indeed, a recent study has reported that the co-inhibition of SHP2 and KRAS in xenograft models is effectively able to suppress feedback reactivation of KRAS pathway, producing a significant tumor shrinkage (35). With regard to immunotherapy, KRAS mutations have been associated with immune checkpoint inhibitors (ICIs) benefit in NSCLC patients. In particular, a subgroup analysis of Checkmate 057 has shown greater outcomes wit ICIs in KRAS-mutant patients (36). Kim et al. (37) compared in a meta-analysis ICIs treatment and docetaxel in pretreated NSCLC patients, stratifying results by KRAS status. They have found that, in KRAS-mutant NSCLC patients, overall survival (OS) was improved with ICI versus chemotherapy; on the other hand, this benefit was not reported in KRAS wild-type patients. Moreover, through the ImmunoTarget registry, it has been evaluated the sensitivity of ICIs in NSCLC patients with a variety of driver mutations. Accordingly, KRAS mutations resulted in increased immunotherapy benefit compared to other driver mutations (38). Recently, new data are available regarding (chemo)immunotherapy efficacy according to KRAS status. Sun et al. have demonstrated that among patients with PD-L1>50% treated with ICI, KRAS mutant tumors are associated with a superior overall survival compared with KRAS wild type (mOS 21.1 *vs* 13.6 months, p=0.03). However, among patients treated with chemoimmunotherapy, there was no significant survival difference between patients with KRAS mutant and KRAS wild type status (mOS, 20.0 *vs* 19.3 months; p=0.93). Moreover, among patients with KRAS mutant status, OS did not differ between those treated with ICI monotherapy and chemoimmunotherapy (mOS, 21.1 *vs* 20.0 months; p= 0.78). The authors conclude that among patients with PD-L1 expression of 50% or greater NSCLC treated with ICI monotherapy, KRAS wild type is associated with worse survival compared with KRAS mutant patients; in contrast, survival did not differ appreciably between patients with KRAS mutant and KRAS wild type status who were treated with chemoimmunotherapy. Definitely patients with PD-L1>50% and KRAS-mutant NSCLC had favorable survival (mOS ≥20 months) with either ICI monotherapy or chemoimmunotherapy, while patients with KRAS wild type who were treated with ICI monotherapy had worse survival compared with those treated with chemoimmunotherapy (39). With regard to KRAS mutation subtype, another study has shown that treatment outcomes with chemoimmunotherapy are similar in patients with G12C and non-G12C subtypes (40). All these results could have a potential biological rationale because of the correlation between a higher incidence of KRAS mutations in smokers and, as already known, the elevated rate of tumoral DNA mutations and the higher tumor mutational burden (TMB) in smoker patients compared to never-smokers (41, 42). Another important issue is the potential clinical relevance of KRAS mutations in combination with other genetic alterations. In particular, variations in KRAS mutation subtypes have been associated with distinct biological behaviors that may led to different clinical outcomes (43, 44). For example, tumors with KRAS G12C mutations exhibited higher ERK1/2 phosphorylation than those with KRAS G12D (45); to support this data, a recent study using mouse models with KRAS-mutant tumors, demonstrated higher efficacy of the MEK inhibitor selumetinib in KRAS G12C cancers compared with KRAS G12D tumors. Concerning additional alterations, EGFR/ALK/BRAF co-mutations are rare in KRAS-mutant lung adenocarcinoma; concomitant mutations in onco-suppressor genes, especially TP53, STK11 and KEAP1, are more often found (11, 14, 46, 47). Furthermore, additional alterations may affect the immunogenicity of KRAS-mutant tumors. For example, it has been recently demonstrated that tumors carrying KRAS/TP53 mutations have an enhanced tumor inflammation, increased PD-L1 expression and tumor-infiltrating lymphocytes (TILs) and, consequently, there is a remarkable clinical benefit wit ICIs (48). In contrast, additional STK11 mutations in KRAS-mutant NSCLC could decrease immune surveillance through NF-kB pathway modulation. Indeed, these tumors are associated with low TILs infiltration that further reduces immune surveillance (49, 50). Several trials have shown that STK11/LKB1 mutations in KRAS-mutant NSCLC might be predictive of primary resistance to ICIs or chemotherapy as well (51–53). Taken together, all this evidence suggests that KRAS mutations cannot be evaluated as a single entity, but it needs to be placed in the context of the specific tumor features and seen as part of a complex process in cancer development and progression. The deeper understanding of underlying KRAS biology and function has allowed, after many years of failures, to develop agents able to stop the winding path towards KRAS unstoppable tumors (**Tables 1**, **2**).

**Table 1:** Trials evaluating KRAS as a prognostic marker.

FIRST author	Type of study	N° of patients (KRAS^mut^ *vs* KRAS^wt^)	Outcome
***Johnson MC***	Retrospective	241 *vs* 520	mOS 16 *vs* 23 m (HR 1.21, p=0.048)
***Mascaux C***	Meta-analysis	625 *vs* 2968	HR 1.35, p=0.01
***Marabese M***	Retrospective	60 *vs* 187	mOS 10.6 *vs* 14.3 m (HR 1.41, p=0.032)
***Shepherd FA***	Pooled analysis	300 *vs* 1246	HR 1.17, p=0.12
***Macerelli M***	Retrospective	39 *vs* 69	mOS 10.3 *vs* 13.2 m (p=0.4) DCR 76% *vs* 91% (p=0.03)

mOS, median overall survival; DCR, disease control rate.

**Table 2:** Trials evaluating KRAS as a potential predictive marker.

FIRST AUTHOR	Type of study	N° of patients (TOT/KRAS^mut^)	Drug	Outcome
***Mao C***	Meta-analysis	1470/231	EGFR-TKI	ORR 3% *vs* 26% (KRAS mut *vs* wt)
***Linardou H***	Meta-analysis	1008/165	EGFR-TKI	Significantly lower ORR in KRAS mut
***Sun JM***	Retrospective	484/39	Pemetrexed	ORR 14% *vs* 28% (KRAS mut *vs* wt)
***Renaud S***	Retrospective	-/1190	CT	Best ORR with taxanes compared to pemetrexed
***Borghaei H***	Retrospective	-/62	Nivolumab *vs* Docetaxel	HR 0.52 (0.29-0.95)
***Kim JH***	Meta-analysis	-/138	ICI *vs* docetaxel	HR 0.64 (0.43-0.96)
***Mazieres J***	Retrospective	551/271	ICI	ORR higher in KRAS compared to other driver mutations
***Sun L***	Retrospective	1127/573	ICI CT + ICI	HR, 0.77 (0.61-0.98) KRAS mut *vs* wt HR, 0.99 (0.70-1.40) KRAS mut *vs* wt

ORR, objective response rate; TKI, tyrosine kinase inhibitor; CT, chemotherapy; ICI, immune-checkpoint inhibitor; wt, wild-type.

## The Era of KRAS Direct Inhibition

Although KRAS was one of the earliest oncogenic drivers discovered, for decades a therapeutic breakthrough in its targeting has been hampered by several biochemical obstacles (such as the high affinity of GTP for KRAS, the difficulty of finding pockets on KRAS large enough for small-molecule drugs), prompting the increasingly widespread opinion that KRAS was definitely undruggable (54, 55). Therefore, tackling KRAS alterations has been focused on inhibition of its membrane binding (or subcellular localization), identification of lethality partners and on inhibition of its downstream effectors. Concerning direct inhibition, the peculiar crystal structure of the KRAS G12C subtype and the unique reactivity of the cysteine thiol result in the ability to covalently bind the cysteine residue within the region adjacent to the nucleotide-binding pocket, inducing a significant perturbation of protein functional domains and a negative allosteric regulation of RAS signaling in tumor cells (56). This feature can be exploited to create covalent small-molecule inhibitors, binding to KRAS and locking cells in the inactive GDP-bound state (57–59). Thus, various pharmaceutical companies have begun to develop KRAS G12C-specific inhibitors, many of which are being explored in ongoing clinical trials. AMG 510/Sotorasib was the first compound to enter clinical trials with promising results since early trials phases, especially in NSCLC patients. Canon et al. (60) have identified this highly selective molecule inducing tumor regression in preclinical models. In particular, AMG 510 potently inhibits cellular viability in KRAS G12X cell lines and induces tumor regression in xenograft models (60). Furthermore, AMG 510 has shown synergistic growth inhibitory effects when combined with other inhibitors of protein that regulate KRAS activation (such as MEK, AKT, PI3K and members of EGFR family) or with immunotherapy (60). Preliminary results of AMG 510 clinical activity have been presented at the 2019 American Society of Clinical Oncology meeting (61), showing a very promising activity in a cohort of 34 NSCLC patient harboring KRAS G12C mutation achieving a surprising objective response rate (ORR) of 48% and a disease control rate (DCR) of 96%. Regarding its safety profile, the adverse events (AEs) have been manageable with only 9% of grade 3-4 AEs (mostly anaemia and diarrhoea) (62). The final results from the phase I trial have shown a median progression-free survival (PFS) of 6.3 months and a median duration of response of 10.9 months in NSCLC subgroup (59 patients), with a confirmed ORR in 32.2% and DCR in 88.1% of patients (63). Thus, the trial has confirmed the antitumor activity of AMG 510 in pretreated NSCLC patients carrying KRAS G12C mutation, consistent with preliminary results (64). The phase II CodeBreak 100 trial has validated the power of KRAS inhibition with sotorasib in patients with advanced NSCLC. In the phase II trial sotorasib has now shown a durable ORR of 37.1%, a DCR of 80.6%, a median PFS of 6.8 months and a median OS of 12.5 months, in the face of grade 3-4 toxicities rate, mostly diarrhea and hepatic transaminase elevation (11%) (65, 66). These exciting data have led to the fast-track designation of AMG 510 by Food and Drug Administration (FDA). Another cysteine 12-directed covalent inhibitor is MRYX849/adagrasib. Similar to sotorasib, adagrasib is a KRAS G12C inhibitor that has demonstrated a great activity on tumor regression in cell lines and patient-derived xenograft models, as well as *in vivo*, showing tumor regression in 65% of KRAS G12C mutant preclinical models including different tumor histotypes (67). Indeed, this agent is under investigation in the KRYSTAL trial, a phase I/II multiple expansion cohort trial of MRTX849 in patients with advanced solid tumors carrying KRAS G12C mutation. In this trial, 79 patients were treated with adagrasib 600 mg BID. Overall, the treatment has been well tolerated and most patients has experienced grade 1-2 AEs, such as nausea, diarrhea, vomiting, fatigue and increased ALT. The only commonly reported (>2%) grade 3-4 treatment-related serious AE has been hyponatremia. Among the 51 patients evaluable for clinical activity, the trial has reported an ORR of 45% and DCR of 96% (68). Interestingly, other studies have highlighted that MRTX849 has synergistic effects when combined with EGFR, Src homology 2 domain-containing tyrosinephosphatase-2 (SHP2), mammalian target of rapamycin (mTOR) or cyclin-dependent kinase 4 (CDK4) and CDK6 inhibitors and its efficacy has resulted increased in case of SHP2, MYC or mTOR gene loss (67). A third KRAS G12C covalent inhibitor is JNJ-74699157 (ARS-3248), currently evaluated in a phase I clinical trial enrolling different KRAS G12C mutated tumor types (NCT04006301). A fourth KRAS G12C covalent inhibitor is LY3499446, evaluated in a phase I/II clinical trial (NCT04165031): the study was early terminated due to an unexpected toxicity finding. Concerning pan-RAS inhibitors, many compounds have been tested but the major issue reported is the difficult discrimination between active and inactive KRAS state, with a consequent high off-target activity and a marked toxicity (69). Regarding preclinical evidence, two previous compounds (ARS-853 and ARS-1620) have demonstrated to reduce cell growth and inhibit downstream signaling to MAPK exclusively in tumor cell lines with KRAS G12C mutations (57, 58, 70). Notably, ARS-853 is a potent G12C allele-specific inhibitor that reduces cancer cell proliferation *in vitro*; its mechanism exploits the need of KRAS G12C nucleotide exchange to switch in the activated status, trapping it in the GDP-RAS state (58, 70). However, ARS-853 has not entered clinical evaluations in KRAS mutant lung cancer yet. Instead, ARS-1620, a covalent compound with high potency and selectivity for KRAS G12C, have shown rapid and sustained tumor regression *in vivo* (57). The interesting feature of tumor cells harboring KRAS mutations is that they develop, over time, escape mechanisms to break free from the KRAS activation status and undergo adaptative changes to became insusceptible to the drug (71). Instead of relying on the drug-inhibited MEK pathway to proliferate, these adapted cells are capable of using EGFR and aurora kinase downstream signaling cascades to remain in the active form (35). These data may help elucidating why most patients enrolled in clinical trials tend to exhibit partial responses to KRAS inhibitors. For this reason, overcome treatment resistance by developing combination therapies might be a promising approach for the future to create a more durable response to KRAS G12C inhibitors (**Table 3**).

**Table 3:** KRAS G12C direct inhibitors.

TRIAL	Phase	Drug	Setting	N° Patients	Results	AE
***CodeBreaK100***	I/II	Sotorasib	≥2L	59	ORR 32.2%, DCR 88.1%, mPFS 6.3m, mOS 10.9m	9% G3/4 (anemia, diarrhoea)
***KRYSTAL-1***	I/II	Adagrasib	≥2L	79	ORR 45%, DCR 96%	G3/4 common TRAE: Hyponatremia (3%)
***NCT04006301***	I	ARS-3248	≥2L		Ongoing	
***NCT04165031***	I/II	LY3499446	≥2L		Closed due to toxicity	

ORR, objective response rate; DCR, disease control rate; mPFS, median progression free survival; mOS, median overall survival; AE: adverse events; TRAE, treatment related adverse event.

### KRAS Indirect Inhibition

Due to the long-standing belief that KRAS was undruggable for the above-mentioned reasons, a large branch of research has been conducted on indirect inhibition. These strategies, which turned out to be mainly unsuccessful, were aimed at KRAS expression (including epigenetic modulation), processing and downstream signaling inhibition.

#### Inhibitors of the Nucleotide Exchange Cycle

Guanine nucleotide exchange factors (GEFs), such as SOS, are essential to promote KRAS signaling by catalyzing the exchange of GDP with GTP, thus resulting in the active KRAS-GTP form; GEF inhibitors are a field of particular interest because their activity is not dependent on the type of KRAS mutation, resulting in pan-RAS inhibition. Several small molecules inhibiting SOS have been identified, although only one has made its way to the clinical experimentation (72). BAY-293 is a SOS1 inhibitor which showed a high affinity for its target but weak activity on cell proliferation *in vitro* (73). BI-1701963 is another SOS1 inhibitor with promising pre-clinical activity, currently tested in a phase I trial, alone or in combination with trametinib, in solid tumors harboring KRAS mutation (NCT04111458).

An interesting new role has been proposed for miR-148a-3p, a tumor suppressor microRNA which controls cell proliferation by reducing the expression of SOS2; in KRAS-mutated cells this microRNA is commonly lost, representing a possible new target in KRAS-mutant NSCLC (74).

Src homology region 2 (SH2)-containing protein tyrosine phosphatase 2 (SHP2) is essential for the activation of KRAS (and other RTKs) signaling, in fact previous studies identified SHP2 as a scaffold protein that links SOS to GRB-2 enhancing the nucleotide exchange cycle (75). Different SHP2 inhibitors are currently under investigation in clinical trials, alone or in combination with other agents; as SHP2 is also implicated in acquired resistance to KRAS G12C inhibitors, there is a strong rationale in combining KRAS and SHP2 inhibitors (71). RMC-4630 has shown promising activity as a single agent in an ongoing phase I clinical trial (NCT03634982), with a DCR of 71% in the cohort of 7 patients with NSCLC and KRAS G12C mutation; RMC-4630 is also being tested in a phase Ib/II clinical in combination with cobimetinib/osimertinib (NCT03989115); JAB-3068 and JAB-3312 are SHP2 allosteric inhibitors investigated in a phase I/II clinical trial (respectively NCT03518554, NCT03565003 and NCT04121286, NCT04045496); TNO155 is being explored as a single agent or with an anti-EGFR (EGF816) in a phase I clinical trial (NCT03114319), in combination with adagrasib in a phase I/II clinical trial (NCT04330664) and in combination with spartalizumab (anti-PD-1) or ribociclib in a phase Ib trial (NCT04000529); RLY-1971 and BBP-398 as single agents are under evaluation in phase I trials (NCT04252339 and NCT04528836).

#### Inhibitors of RAS Post-Translational Processing

KRAS undergoes several post-translational modifications in order to become active (76). The first step is the prenylation of the carboxyl terminal by the addition of a farnesyl tail, mediated by farnesyltransferase (FTase) or geranylgeranyltransferase (GGTase). Although an interesting preclinical activity of FTase inhibitors (FTIs), first-generation FTIs tipifarnib and lonafarnib, and second-generation salirasib, have not shown significant activity in NSCLC patients (77–79). A possible mechanism of intrinsic resistance to FTIs may be the alternative prenylation of KRAS by GGTase; preclinical data regarding the activity of FGTI-2734, a dual FTase and GGTase inhibitor, on KRAS mutant cell lines (including a NSCLC line) are promising (80). Another potential target involved in KRAS post-translational modifications comprehends phosphodiesterase-δ (PDE-δ), a prenyl-binding protein that transports prenylated KRAS to the cellular membrane; NHTD is a PDE-δ inhibitor with promising *in vitro* and *in vivo* activity on mouse xenografts (81).

#### Inhibitors of Downstream Signaling

Targeting KRAS intracellular pathways has not scored interesting results due to the redundancy and crosstalk of downstream signaling. The inhibition of RAF/MEK/ERK pathway using selective BRAF inhibitors, such as vemurafenib or dabrafenib, whose activity is well established in tumors carrying BRAF V600E mutation, is not effective in KRAS mutant tumors. These drugs can effectively bind only to BRAF monomers, but in KRAS mutant tumors the MAPK pathway is activated by RAF dimers, which are not inhibited by vemurafenib or dabrafenib; in addition, these TKIs cause an upregulation in ERK phosphorylation and activation mediated by CRAF (the so called “MAPK paradox”) (82). On the other hand, nonselective RAF inhibitors like sorafenib (multi-TKI inhibitor of RAF, VEGFR and PDGFR amongst others), did not show a relevant clinical activity in subgroups of KRAS mutant NSCLC: in the MISSION phase III trial sorafenib was tested against placebo in pretreated NSCLC patients, and although a significantly longer PFS, OS was similar between the two arms both in the overall population (8.2 *vs* 8.3 months; HR, 0.99, p=0.49) and in the KRAS mutant population (6.4 *vs* 5.1 months; HR, 0.76, p=0.279) (83); in the BATTLE-1 and BATTLE-2 trials, 8-weeks DCR was non statistically different with sorafenib according to KRAS status, although in the latter study RAF and MEK inhibitors showed a better clinical activity than EGFR inhibitor erlotinib in KRAS mutant patients (84, 85). MEK inhibitors have been used alone or in combination with chemotherapy, based on preclinical data of enhanced activity, with disappointing results up to now. The randomized phase III SELECT-1 trial has compared selumetinib plus docetaxel versus docetaxel in 510 patients with KRAS mutant pretreated NSCLC, showing no significant difference in mPFS (3.9 *vs* 2.8 months, p=0.44) and mOS (8.7 *vs* 7.9 months, p=0.64) (86). Trametinib has not demonstrated a better clinical activity compared to docetaxel in a randomized phase II study conducted on KRAS mutant NSCLC patients (87); trametinib has also been evaluated in phase I/Ib trials in combination with docetaxel or pemetrexed in patients with NSCLC, showing a favorable toxicity profile and a slightly better activity on patients with KRAS mutation compared to KRAS wild-type patients (88, 89). Binimetinib has been tested in the first-line setting in the phase IB SAKK 19/16 study, in combination with platinum-based chemotherapy, in patient with KRAS mutant NSCLC; the addition of binimetinib has not shown early signs of clinical activity (90). Several trials are being conducted using MEK inhibitors alone or in combination with other agents. A preclinical study by Lee et al. has shown that in murine models of KRAS G12D/p53- lung cancer, the combination of trametinib with anti-PD-1 or anti-PD-L1 immune-checkpoint inhibitors has synergistic anti-tumor effect; this might be explained by an immunomodulatory effect of trametinib that, according to the authors, might be determined by the depletion of PMN-MDSC from the tumor microenvironment (91). ERK inhibitors have shown little activity as monotherapy, so they are currently under investigation mostly in combination with other drugs (e.g. ICIs or other targeted agents) in phase I basket trials (NCT03745989; NCT04418167; NCT03415126).

PI3K-AKT-mTOR is another downstream pathway whose signaling is started by the phosphorylation of PI3K by means of KRAS-GTP; it has important interconnections with RAF-MEK-ERK pathway, in fact the inhibition one of them causes a compensating hyperactivation of the other (92). This might be the reason why single agents directed against this pathway have proven to be mostly unsuccessful. In the phase II BASALT-1 trial, buparlisib, a pan-PI3K inhibitor, was tested in a cohort of 63 patients with pretreated NSCLC harboring a PI3K pathway activation, including 3 patients with concomitant KRAS alterations; the study was closed due to futility, with a 12-week PFS of 23.3% and 20% in squamous and non-squamous histology (93). After the disappointing results of everolimus in unselected patients with pretreated NSCLC (RR<5%), a phase II trial with ridaforolimus was conducted on 79 patients with pretreated KRAS mutated NSCLC, obtaining similar outcomes (RR 1%) (94). More emphasis should be given to combination therapies which include PI3K pathway inhibitors, as it is involved in resistance mechanisms to direct KRAS inhibitors: preclinical studies have demonstrated that the addition of PI3Ki to ARS1620, a selective RAS G12C inhibitor, was effective in cell lines and in xenografts after the failure of ARS1620 monotherapy (95). Dual inhibition of MAPK and PI3K pathways has shown synergistic effects on cell proliferation in preclinical models (96–98), but in the clinical setting alarming signs of toxicity have been reported (99, 100).

#### Synthetic Lethality

Synthetic lethal screenings are used to identify, in different cell lines, genes that are indispensable for cell survival and proliferation. In KRAS mutated cell lines, previous studies have shown that selective inhibition of BCL-XL, CDK4, XPO1, GATA2, or NF-κB results in cell death, according to the principle of synthetic lethality (101–105). Abemaciclib, a CDK4/6 inhibitor, has been investigated in the JUNIPER trial, a phase III study in KRAS mutated pretreated NSCLC, and it has not improved OS compared to erlotinib, the control arm (7.4 *vs* 7.8 months, HR 0.968, p=0.77) (106). Although palbociclib, another CDK4/6i, hasn’t shown relevant clinical activity in patients with pretreated NSCLC harboring cell cycle gene alterations (107), preclinical data suggest that the combination of palbociclib and MEKi has synergistic antitumoral and radiosensitizing effects on KRAS mutated NSCLC cell lines (108, 109); different clinical trials are evaluating this combination in the clinical setting (NCT03170206; NCT02022982). Based on synthetic lethality, NCT02079740 is a phase Ib/II trial investigating safety and tolerability of the combination of the BCL inhibitor navitoclax and trametinib, while NCT03095612 is a phase I/II trial evaluating selinexor, an inhibitor of exportin-1 (XPO1), in combination with docetaxel in previously treated KRAS mutant NSCLC. NF-κB is a key transcription factor for KRAS mutated NSCLC; its activity is downregulated by the proteasome inhibitor bortezomib, which restores the function of IκB (NF-κB inhibitor). In a phase II trial, 16 patients with previously treated NSCLC harboring KRAS G12D mutation were treated with bortezomib, but results were disappointing: only 1 partial response was seen, mPFS was 1 month and mOS 13 months. To note, the patient that obtained a partial response had a special histotype (invasive mucinous adenocarcinoma) (110).

#### New Potential Approaches

Although the recent discovery of direct inhibitors, KRAS mutated NSCLC remains a challenging disease. Several mechanisms of resistance to direct inhibitors have been identified, both on-target (acquired point mutations or KRAS amplification) and off-target (activating mutations in RAF/MEK/ERK pathway, oncogenic fusions of other oncogenes such as ALK and RET or histological transitions) (111). Different strategies to improve the results obtained so far are under investigation, for example the use of KRAS direct inhibitors in combination with immunotherapy in the phase II KRYSTAL-7 trial (NCT04613596) and in the phase Ib CodeBreak 101 trial (NCT04185883). Recently, preliminary results about two combination arms of the CodeBreak 101 trial were presented at the AACR-NCI-EORTC 2021 Virtual International Conference on Molecular Targets and Cancer Therapeutics. In 33 pretreated patients with KRAS-mutant NSCLC, sotorasib was combined with two different doses of afatinib (20 mg in cohort one and 30 mg in cohort two), showing respectively an ORR of 20% and 35% and a DCR of 70% and 74%; the most common grade 3 AE was diarrhea. Sotorasib and trametinib combination was tested in 18 patients with heavily pretreated NSCLC, achieving early signs of activity: DCR was 87% in patients not previously treated with a KRAS G12C inhibitor; the most common AEs were diarrhea, nausea, vomiting and skin reactions (112). Other fields of research are exploring new potential indirect targets, for example p65BTK, an isoform of Bruton’s tyrosine kinase expressed in more than 50% NSCLC samples that acts as a downstream effector of RAF/MEK/ERK pathway in KRAS mutated cell lines; this important oncogene, which up to now has been known to be expressed only in hematopoietic cells, could be targetable by BTK inhibitors like ibrutinib (113). A further important open issue is targeting non-G12C KRAS mutations; for instance, KRAS G12V mutation, although more common in colorectal cancers (CRC) or pancreatic adenocarcinomas, accounts for about 6% of NSCLC mutations. Direct KRAS G12V inhibitors are still in the preclinical setting, but they showed promising results in patient-derived xenograft models of NSCLC, CRC and pancreatic cancers, obtaining even complete tumor regressions (114). The deeper understanding of the metabolic reprogramming in KRAS-mutant cells has allowed to identify additional vulnerabilities. Kerr and colleagues showed that KRAS G12D copy gain, which is a common step during tumor progression, not only induces a glycolytic switch in lung cancer models, but also enhances glutathione (GSH) biosynthesis, translating into lower ROS levels and increased resistance to oxidative stress; treating KRAS-mutant NSCLC cell lines with the glucose analogue 2-deoxy-D-glucose and with BSO (an inhibitor of the synthesis of GSH) resulted in a dramatic apoptosis, confirming their dependency on glucose and GSH (115). The upregulation of fatty acid synthetase (FASN) is also frequent in KRAS-mutant cancer cells in order to provide a large amount of lipids, which are necessary to store energy and build cell membranes (116); indeed, FASN inhibitor TVB-2640 was tested (alone or in combination with a taxane) in a phase Ib trial in solid tumors including a cohort of pretreated NSCLC patients: a higher activity was seen in patients harboring a KRAS mutation, with a median time to progression of 22 weeks *vs* 5 weeks (p<0.02) (117). Lastly, LKB1 mutations are found simultaneously with KRAS mutation in about 10% of NSCLC and seems to confer susceptibility to phenformin, a mitochondrial inhibitor and analogue of the antidiabetic drug metformin. This might be explained because LKB1 is a kinase responsible for the phosphorylation of AMPK under condition of energy stress (low intracellular ATP levels), leading to the control of cell growth; in the absence of LKB1, the depletion of ATP induced by phenformin results in apoptosis due to the inability of these cells to recognize the state of energy deficiency (118).

### Epigenetic KRAS Modulation

Epigenetic modulation of gene expression is an important regulatory process in biology (119) and its alterations contribute to cancer development and progression. Gene regulation occurs in the context of packaging of DNA into an organizing structure, the nucleosome, composed of a DNA strand wound around a core of eight histone proteins (120). Each histone protein has a N-terminal tail extended outward through the DNA strand. Amino acid residues on the histone tail are modified by post-translational methylation, acetylation and phosphorylation (121). On the contrary, deacetylation, demethylation, and dephosphorylation of histones work decreasing access of transcription factors to promoter regions (122). These post-translational modifications can alter the secondary structure of the histone protein tails, increasing the distance between DNA strands and histones and facilitating the access to transcription factors to gene promoter regions (123). Therefore, it is clear that epigenetic regulation is strongly correlated with gene expression and, potentially, with cancer development. In particular, epigenetic modification concur to lung cancer spreading (124–126) and some data available have shown that there is an interaction between KRAS mutations and epigenetic changes (127). For example, histone deacetylase inhibitors have shown the ability of blocking KRAS signalling through gene transcription inhibition and by promoting apoptosis process. Indeed, promising results have been achieved for the treatment of several tumours (122). Notably, Panobinostat has been evaluated in KRAS-mutant NSCLC A549 cell line, demonstrating a significant reduction of cell proliferation (128). Yamada et al. (129) have also evaluated the synergistic efficacy of MEK plus a histone deacetylase inhibitor, demonstrating that this combination has activity in KRAS-mutant lung cancer cells and might be consider for a promising novel therapeutic approach for patients with NSCLC harbouring KRAS mutations. Liu et al. (127) have demonstrated that KRAS mutations increase telomerase activity and length using RAS/MEK pathway activation. *In vitro* experiments have shown that there is a clear correlation between chemoresistance in KRAS-mutant tumors and telomerase length; as consequence, targeting telomerase/telomere may represent a potential therapeutic strategy for patients carrying KRAS mutations. Unluckily, imetelstat, a telomerase inhibitor, has been tested in a phase II study as maintenance treatment and has failed to improve PFS in patients with advanced NSCLC, only showing a trend toward an improved PFS and OS in patients with shorter telomere (130). Hong et al. (124) have speculated that GSK-J4, the histone H3K27 demethylase inhibitor, is able to induce oxidative and metabolic stress in cancer cell lines when a KRAS mutation is detected, linking the role of oncogenic KRAS in the metabolic stress response to GSK-J4 sensitivity. The trial results suggest GSK-J4 as a potential treatment option for cancer patients with KRAS mutations. Indeed, previous studies have shown that KRAS mutant cancers are more sensitive to oxidative stress (131, 132); moreover, a recent study has exhibited that in NSCLC with KRAS mutations there is an altered expression of genes involved in metabolism upon glutamine deprivation, confirming the relationship between KRAS mutations and oxidative stress susceptibility (133). Sunaga et al. (134) have used RNA interference (RNAi) to knock down the KRAS-mutant cells transcript. They have demonstrated that this mechanism reduces cell proliferation through MAPK pathway downregulation, but cancer progression is not completely abolished due to escape pathways using EGFR, STAT3 and Akt phosphorylation. These findings reveal that inhibition of KRAS signaling is effective, but it could be not enough for a such complex and sophisticated issue (134). Another study has examined the inhibitory growth effects of an anti-KRAS ribozyme adenoviral vector (KRbz-ADV) in *in vivo* and *in vitro* NSCLC models. In this study, KRbz-ADV has reduced NSCLC cell growth, determining tumor shrinkage *in vitro* and inducing complete xenograft regression in 70% of cases after repeated intratumoral injection of KRbz-ADV *in vivo* (135). A further epigenetic regulation is mediated by bromodomain and extraterminal (BET) proteins that bind acetylated histones and recruit transcription factors to active promoters and enhancers. For this reason, agents targeting BET proteins have emerged as potential therapeutic strategies (136) and have been tested in hematopoietic and solid tumors, showing a significant MYC suppression in cancer cells (137). Interestingly, BET inhibitors have demonstrated to suppress KRAS pathway, in particular in NF1-mutant tumors, by suppressing KRAS-driven transcriptional output (138); as consequence, MEK and ERK downstream appear silenced. Therefore, Guerra et al. have demonstrated that BET inhibitors might enhance the efficacy of MEK inhibitors in KRAS-mutant cancers (139). They have also identified a subset of lung cancer harbouring KRAS mutations. Specifically, several tumors express high level of the homeobox gene HOXC10. HOX genes are development controllers often overexpressed in cancer and, on the contrary, are normally expressed in the corresponding tissue during non-tumoral development (139, 140). In the case of lung tissues, HOX genes are not expressed at all during lung development, on the other hand HOXC10 appears overexpressed in NSCLC. In this study, the authors have shown that HOXC10 is a biomarker of response to MEK/BET inhibitors and regulates expression of pre-replication complex proteins in conjunction with MEK. Thus, MEK/BET inhibitor can suppress KRAS output and HOXC10 to trigger replication defects and induce cell death (139). Fabrizio et al. have investigated the role of methylation of Kelch-like ECH-associated protein cytosine-guanine dinucleotide (KEAP1CpGs); they have found that this methylation shows a significant inverse correlation with the KEAP1 transcript levels. Notably, these results were limited to the KRAS wild-type squamous cell carcinoma and adenocarcinoma, whereas in adenocarcinoma histotype the effect of epigenetic silencing of KEAP1 was also observed in the EGFR mutated subpopulation. In conclusion, the study has revealed that the epigenetic regulation of KEAP1 expression has different features in KRAS and EGFR settings of NSCLC, suggesting KEAP1 methylation as a predictive marker for response to anti-EGFR agents in oncogene addicted disease. Moreover, the correlation between epigenetic features and histotype underlies an interplay with lung cancer pathogenesis and development (141). Despite the many obstacles and its intrinsic complexity, the intriguing scenario of epigenetic targeting has led to many efforts trying to find the weak spot of KRAS-mutant tumors, although in this regard there is still a long way to go.

## Conclusions

**Figure 1:**
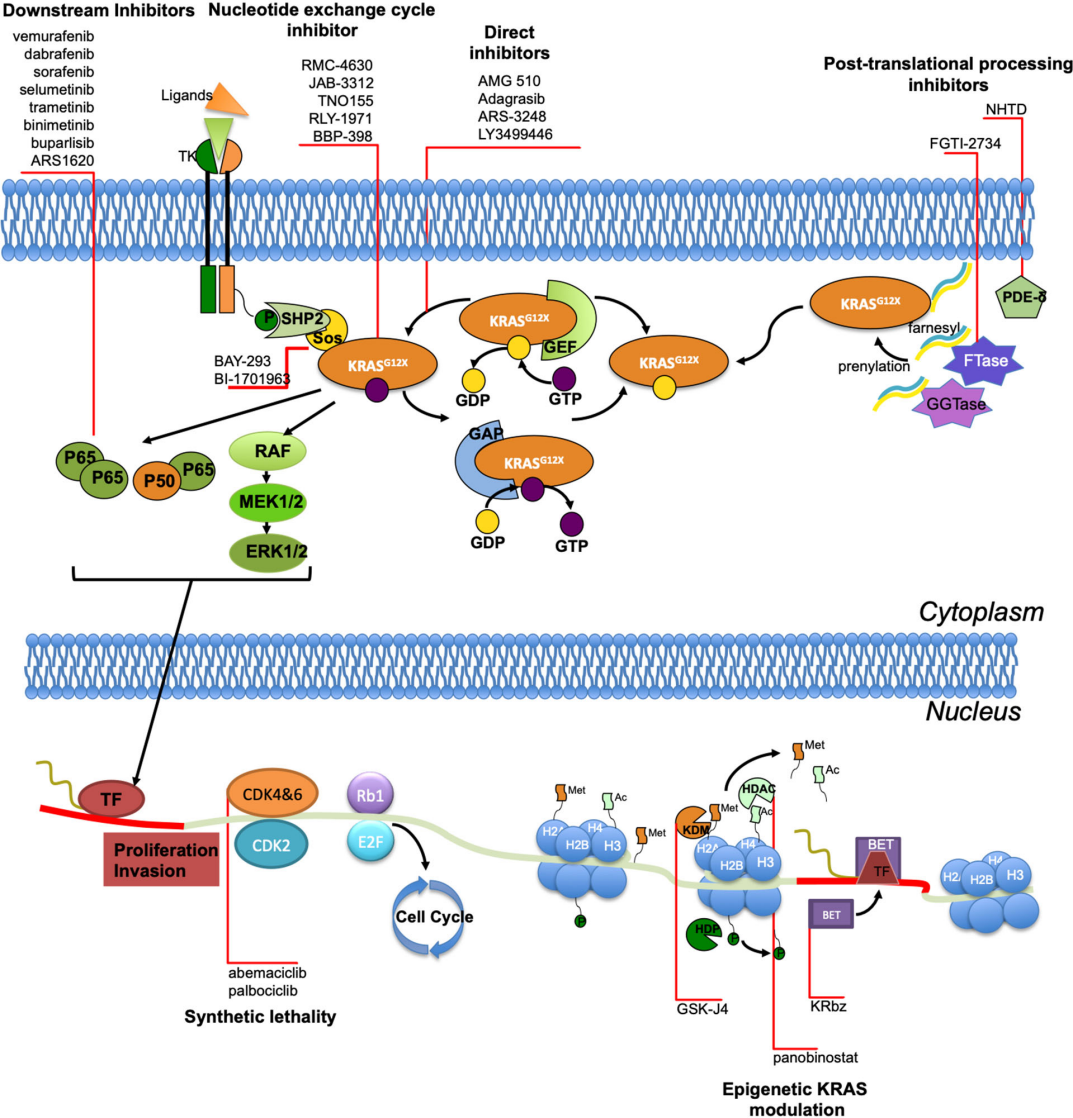
Site of activity of different agents targeting KRAS.

NSCLC is a heterogeneous disease, in which the only histological classification is largely inadequate to completely define its intrinsic complexity. Molecular markers have dramatically reshaped NSCLC treatment, but KRAS mutations still represent a challenge for physicians and patients. Recently, promising compounds have opened the doors to a new era in which KRAS targeting is possible. The most promising therapeutic approaches are represented by KRAS G12C direct inhibitors, AMG 510 and MRTX849, that have shown encouraging preliminary results in ongoing clinical trials. Actually, the impressive preclinical and clinical activity showed by AMG 510, has led to the fast-track designation by the FDA, representing one of the major breakthrough in the lung cancer research of the last years (142). Other treatment strategies including the inhibition of specific pathways, such as RAF/MEK/ERK, are under investigation as well (NCT03745989; NCT04418167; NCT03415126). The role of ICIs in KRAS mutant NSCLC treatment is also under consideration, alone or in combination with targeted therapy approaches (NCT04613596). Also epigenetic deregulation has been increasingly recognized as one of the major mechanisms of gene expression modulation in lung cancer. Furthermore, some data support the hypothesis that there could be an interplay between KRAS epigenetic regulation and lung cancer pathogenesis (141), suggesting a sophisticated balance between genomic and epigenomic features and lung cancer development. However, although much progress has been made in KRAS-mutant NSCLC treatment, clinical trials have shown that outcomes cannot be given for granted even when the same subtype mutation occurs. This limited efficacy underlies a relevant molecular heterogeneity among KRAS-mutant lung cancer, with such an innate or acquired resistance to targeted therapy, requiring further investigations. Overcoming mechanisms of resistance, implementing gene expression profiling in routine practice, tailoring treatment strategies and extending pharmacological approaches to KRAS mutations other than G12C currently represent the biggest challenge to be addressed for the near future. On the whole, the historical undruggable KRAS alteration is likely to be targeted and a significant turning point has been reached, allowing to offer a range of opportunities in the growing individualized treatment paradigm for KRAS-mutant NSCLC patients (**Figure 1**).

## Author Contributions

MF, AS, and EB, conceived the original idea of the article, drafting, and writing the paper. CC, SP, LB, MM, and GP revised the scientific content of specific sections of the manuscript and participated in drafting specific section of the paper. EV, MD, IS, FM, AV, GV, and ED, participated in the critical revision of the paper. MM, GT, and EB participated in the critical revision and editing of the manuscript. GT and EB conceived the original idea and provided critical revision of the manuscript as well as the final approval of the version to publish. All authors contributed to the article and approved the submitted version.

## Funding

EB is supported by Institutional funds of Università Cattolica del Sacro Cuore (UCSC-project D1-2019-2021). EB is currently supported by the Associazione Italiana per la Ricerca sul Cancro (AIRC) under Investigator Grant (IG) No. IG20583. GT is supported by AIRC under IG No. IG18599. CC is supported by AIRC Grant “Luigi Bonatti and Anna Maria Bonatti Rocca”.

## Conflict of Interest

EB received speakers’ and travels’ fee from MSD, Astra-Zeneca, Pfizer, Eli-Lilly, BMS, Novartis and Roche. EB received institutional research grants from Astra-Zeneca, Roche.

The remaining authors declare that the research was conducted in the absence of any commercial or financial relationships that could be construed as a potential conflict of interest.

## Publisher’s Note

All claims expressed in this article are solely those of the authors and do not necessarily represent those of their affiliated organizations, or those of the publisher, the editors and the reviewers. Any product that may be evaluated in this article, or claim that may be made by its manufacturer, is not guaranteed or endorsed by the publisher.
